# Exploratory observational study with Two-year outcomes of early in-hospital evolocumab in acute coronary syndrome patients undergoing coronary artery bypass grafting

**DOI:** 10.3389/fcvm.2026.1705964

**Published:** 2026-03-05

**Authors:** Giuseppe Nasso, Walter Vignaroli, Giuseppe Santarpino, Claudio Larosa, Isabella Rosa, Francesco Bartolomucci, Vincenzo Montemurro, Flavio Fiore, Antongiulio Valenzano, Giacomo Errico, Giacomo Schinco, Mario Siro Brigiani, Gaetano Contegiacomo, Vito Margari, Michele Covelli, Alfredo Marchese, Maria Antonietta De Mola, Ernesto Greco, Giuseppe Speziale

**Affiliations:** 1 Department of Cardiac Surgery, Santa Maria Hospital and Anthea Hospital GVM Care & Research, Bari, Italy; 2 Department of Medicine and Surgery, LUM University, Bari, Italy; 3 Department of Cardiac Surgery, San Carlo di Nancy Hospital GVM Care & Research, Rome, Italy; 4 Department of Cardiac Surgery, Città di Lecce Hospital, GVM Care & Research, Lecce, Italy; 5 Department of Clinical and Experimental Medicine, Magna Graecia University, Catanzaro, Italy; 6 Department of Cardiac Surgery, Paracelsus Medical University, Nuremberg, Germany; 7 Department of Cardiology, Azienda Ospedaliera B.A.T., Bonomo Hospital, Andria, Italy; 8 Department of Cardiology, CDC Scilla ASP reggio calabria, Calabria, Italy; 9 Department of Health and Life Sciences European. University of Rome, Rome, Italy

**Keywords:** acute coronary syndrome, coronary artery bypass grafting, evolocumab, LDL-C, PCSK9 inhibitor, secondary prevention

## Abstract

**Aims:**

Patients with acute coronary syndrome (ACS) who require coronary artery bypass grafting (CABG) remain at very high ischemic risk due to diffuse native disease and vein graft vulnerability. This study aimed to assess whether very early in-hospital initiation of evolocumab, a Proprotein Convertase Subtilisin/Kexin Type 9 (PCSK9) inhibitor, on top of statins improves cholesterol control and mid-term cardiovascular outcomes in this high-risk population.

**Methods:**

We performed a single-center, retrospective cohort study of 74 ACS patients undergoing isolated CABG (January 2022–July 2023) at Anthea Hospital GVM Care & Research, Bari, Italy. All received high-intensity statin therapy ± ezetimibe (STANDARD, *n* = 43), while 31 also received evolocumab 140 mg every two weeks (EVOLOCUMAB), initiated pre-angiography, preoperatively, or within 72 h post-CABG. The primary endpoints were LDL cholesterol (LDL-C) trajectory and attainment of <55 mg/dL at 24 months, and major adverse cardiovascular events (MACE: cardiovascular death, spontaneous myocardial infarction, or any revascularization).

**Results:**

Seventy-one patients completed 24-month follow-up (EVOLOCUMAB *n* = 30; STANDARD *n* = 41). Baseline LDL-C was similar between groups (∼156 mg/dL). Evolocumab produced rapid and durable LDL-C reduction: at 24 months, mean LDL-C was 52 ± 11 mg/dL (EVOLOCUMAB) vs. 82 ± 18 mg/dL (STANDARD, *p* < 0.001). LDL-C < 55 mg/dL was achieved by 73.3% of EVOLOCUMAB vs. 29.3% of STANDARD patients (*p* < 0.001). MACE occurred in 10.0% (EVOLOCUMAB) vs. 24.4% (STANDARD), with lower risk in EVOLOCUMAB (HR 0.48, 95% CI 0.22–0.94; *p* = 0.035), mainly due to fewer repeat revascularizations. Evolocumab was well tolerated; no discontinuations due to adverse events were observed

**Conclusion:**

In ACS patients undergoing CABG, very-early in-hospital evolocumab plus statins achieved sustained LDL-C lowering and fewer adverse cardiovascular events over two years. Given the retrospective observational design, causal inference is limited and residual confounding cannot be excluded. These findings are hypothesis-generating and require confirmation in randomized trials.

## Introduction

Acute coronary syndrome (ACS) remains a leading cause of morbidity and mortality worldwide and confers a substantial risk of recurrent ischemic events beyond the index hospitalization (1, 2). Despite contemporary pharmacologic and revascularization strategies, residual risk persists for months to years, driven by a high burden of vulnerable plaques throughout the coronary tree and by systemic pro-inflammatory and pro-thrombotic activity (3, 4). In patients with established atherosclerotic cardiovascular disease (ASCVD), intensive low-density lipoprotein cholesterol (LDL-C) lowering consistently reduces major adverse cardiovascular events (MACE), with evolocumab demonstrating additive clinical benefit on top of statins in large outcomes trials and long-term extensions (1, 2, 5–9).

Patients with ACS who require coronary artery bypass grafting (CABG) represent a particularly vulnerable subgroup. They often present with complex multivessel and/or left main disease, diffuse atherosclerosis, and frequent comorbidities such as diabetes or chronic kidney disease (10, 11). Surgical revascularization improves prognosis but does not halt atherosclerosis in native vessels, and saphenous vein grafts (SVGs) are prone to early intimal hyperplasia followed by accelerated atherogenesis, contributing to graft failure within the first years after surgery (10, 11). This combination of diffuse native disease and graft vulnerability sustains an elevated risk of myocardial infarction, repeat revascularization, and cardiovascular death in the mid-term post-CABG period.

Early, aggressive secondary prevention is therefore pivotal. Profound LDL-C reduction on top of high-intensity statins lowers ischemic events in ASCVD, and PCSK9 inhibition with evolocumab typically provides an additional 50%–60% LDL-C reduction (1, 2, 5, 12). The EVOPACS trial demonstrated that very-early in-hospital initiation of evolocumab during ACS is feasible, safe, and rapidly achieves LDL-C targets within weeks, supporting an “early-strike” lipid-lowering strategy (2). European guidelines recommend stringent LDL-C targets (<55 mg/dL for very-high-risk patients, <40 mg/dL in those with recurrent events within two years) and emphasize achieving them as quickly as possible (6), while long-term data confirm durability of LDL-C lowering and sustained safety with evolocumab (7).

Evidence on early PCSK9 inhibition specifically in ACS–CABG patients remains limited. Real-world registries suggest that a “strike-early, strike-strong” strategy is operationally feasible, achieving substantial LDL-C reductions without safety concerns (12). Retrospective analyses in ACS patients undergoing CABG indicate that introducing PCSK9 inhibitors is associated with favorable lipid profiles, although sample sizes were small and initiation timing varied (6). These observations highlight a knowledge gap regarding very-early in-hospital PCSK9 initiation in surgical ACS patients and its potential impact on mid-term outcomes.

The pathophysiologic rationale for early PCSK9 inhibition in ACS–CABG is compelling. The post-ACS period is characterized by pancoronary plaque instability, and rapid LDL-C lowering may stabilize vulnerable plaques and reduce distal events (2, 5). SVGs are particularly susceptible to early intimal hyperplasia and atherogenic remodeling; maintaining very low LDL-C levels soon after surgery could slow graft atherosclerosis and reduce graft-related reinterventions. Evolocumab has demonstrated sustained lipid control with long-term safety (7), and initiating therapy before discharge may minimize delays and therapeutic inertia associated with stepwise intensification (statin → ezetimibe → PCSK9 inhibitor) (6, 7, 12).

This study aimed to evaluate the effectiveness and feasibility of very-early in-hospital initiation of evolocumab on LDL-C target attainment in ACS patients undergoing CABG, and to explore its association with mid-term cardiovascular outcomes. Building on and extending our previously published experience with evolocumab in dyslipidemic patients undergoing coronary artery bypass grafting, the present investigation represents a continuum of our center's ongoing clinical experience in this setting. Against this background, we designed a single-center observational study to evaluate the mid-term effectiveness and safety of early in-hospital evolocumab initiation on top of high-intensity statin therapy in consecutive ACS patients undergoing isolated CABG. We compared patients treated with evolocumab plus guideline-directed therapy (EVOLOCUMAB) to those receiving statin ± ezetimibe alone (STANDARD), assessing LDL-C trajectories and attainment of <55 mg/dL through 24 months, alongside a prespecified composite endpoint of MACE (cardiovascular death, spontaneous myocardial infarction, or any coronary revascularization). We hypothesized that very-early initiation would produce durable LDL-C separation and reduce mid-term ischemic events compared with stepwise statin-based strategies, consistent with mechanistic and clinical evidence for PCSK9 inhibition (2, 5–8, 12–14).

## Methods

We performed a single-center, retrospective, observational cohort study at a tertiary cardiac surgery institution (Anthea Hospital GVM Care & Research, Bari, Italy). Consecutive adults hospitalized with acute coronary syndrome (ACS) who underwent isolated coronary artery bypass grafting (CABG) between January 2022 and July 2023 were screened for inclusion. ACS was defined according to contemporary guideline criteria [ST-elevation myocardial infarction [STEMI], non–ST-elevation myocardial infarction [NSTEMI], or unstable angina] (6). The study conformed to the Declaration of Helsinki and institutional regulations, was approved by the Institutional Review Board/Comitato Etico for retrospective studies (Protocol No. 101.10.2023), and was registered in the Italian Medicines Agency (AIFA) observational registry (ID 781; code AH.011.02.023). Because of the retrospective design and use of deidentified data, the requirement for individual informed consent was waived.

All eligible patients were identified from institutional electronic health records (EHRs) and cardiac surgery databases. Patients were included if they were ≥18 years old, admitted with ACS, and underwent isolated CABG during the index hospitalization. Given the very-high-risk profile of ACS–CABG and the objective of early intensification of lipid lowering, eligibility required a baseline low-density lipoprotein cholesterol (LDL-C) ≥ 100 mg/dL or a clinician-determined very-high-risk status warranting immediate intensification consistent with guideline principles (6). No restrictions were applied regarding the type of CABG procedure; patients undergoing CABG with arterial grafts, venous grafts, or combined arterial and venous grafting were all included in the study.

Patients were excluded if they had prior exposure to a PCSK9 inhibitor; active infection at the time of index admission; severe hepatic dysfunction (alanine or aspartate aminotransferase >3× the upper limit of normal); anticipated noncardiac life expectancy <1 year; or inability/refusal to participate in scheduled follow-up. Patients undergoing concomitant valve surgery or those lacking evaluable 24-month outcomes were not included in the analytic cohort.

Two contemporaneous treatment pathways reflected local policy and drug availability:

1. STANDARD (standard care): high-intensity statin therapy (e.g., atorvastatin 40–80 mg or rosuvastatin 20–40 mg) with optional ezetimibe 10 mg at clinician discretion, aligned with guideline-directed care (6).
2. EVOLOCUMAB (early evolocumab): the same background regimen plus evolocumab 140 mg subcutaneously every 2 weeks, initiated during the index hospitalization—preferably before diagnostic angiography; if not feasible, preoperatively or within 72 h after CABG. Dosing and in-hospital timing were consistent with early-initiation strategies explored in ACS (2) and with the efficacy/safety profile established in outcomes programs (2, 5–7). No bridging strategies were used.

Background ACS management (antiplatelet therapy, anticoagulation, beta-blockers, ACEI/ARB/ARNI where appropriate) and perioperative care followed guideline-consistent institutional protocols (6). Statin intensity and ezetimibe use were recorded at discharge and throughout follow-up.

Patients were evaluated at regular 6-month intervals up to 24 months after the index hospitalization (i.e., approximately at 6, 12, 18, and 24 months), either in person or via structured telehealth contacts. At each visit we obtained fasting lipid profiles (total cholesterol, LDL-C, HDL-C, triglycerides) and liver enzymes, and systematically recorded adverse events and medication changes. Medication adherence was assessed by patient report, pharmacy refill history, and the proportion of days covered (PDC); for PCSK9 inhibitors, adherence ≥80% defined high persistence.

No participant initiated bempedoic acid during follow-up and in the STANDARD group, no patients started a PCSK9 inhibitor.

When clinically appropriate and logistically feasible, coronary CT angiography [coronary CT angiography (CCTA)] was encouraged around 24 months to assess graft patency. Graft occlusion on coronary CT angiography (CCTA) was defined as absence of contrast opacification or an abrupt contrast cutoff on multiplanar reconstructions. Vital status was verified through EHRs, institutional registries, and direct contact with patients or next of kin.

### Primary endpoints

1. Laboratory endpoint: LDL-C trajectory and attainment of LDL-C < 55 mg/dL at 24 months, consistent with targets for very-high-risk patients (6).
2. Clinical endpoint (MACE): a composite of cardiovascular death, spontaneous myocardial infarction, or any coronary revascularization (native vessel or graft).

Secondary endpoints included the individual components of MACE; graft-related revascularization; all-cause death; and safety/tolerability metrics (treatment discontinuation, injection-site reactions, clinically significant transaminase elevations). Safety monitoring focused on hepatic enzymes and muscular toxicity in line with the established safety profile of evolocumab (2, 5–7).

Potential clinical events were ascertained from hospital documentation. For revascularization events, procedural reports and angiographic records were evaluated to classify target (native vs. graft). For deaths, cardiovascular causality was adjudicated from clinical records and, when available, death certificates.

Baseline demographics, comorbidities, surgical details, laboratory values, and follow-up data were abstracted from the EHR via a standardized case report form with predefined variable dictionaries. Data integrity checks included range validation, logic rules for temporal consistency, and review of outliers; queries were reconciled against source documents.

### Statistical analysis

Continuous variables are reported as mean ± standard deviation or median [interquartile range]**,** and compared between groups using Student's t test or the Mann–Whitney U test, as appropriate. Categorical variables are summarized as counts (percentages) and compared using χ^2^ or Fisher's exact tests.

Time-to-event outcomes were depicted with Kaplan–Meier curves and compared using the log-rank test. Hazard ratios (HRs) for MACE were estimated with Cox proportional hazards models adjusted for clinically relevant covariates [age, sex, diabetes, baseline left-ventricular ejection fraction (LVEF), baseline LDL-C, and chronic high-intensity statin therapy], selected *a priori* based on clinical judgment and prior literature (2, 5, 6, 8, 12). Proportional hazards assumptions were evaluated with Schoenfeld residuals. Robust (Huber–White) standard errors were applied.

A multivariable logistic regression model identified independent predictors of LDL-C < 55 mg/dL at 24 months (prespecified covariates: treatment group, PCSK9i adherence, baseline LDL-C, age, sex, diabetes, LVEF, high-intensity statin) (Figure 2B). Collinearity was assessed via variance inflation factors. Missing laboratory data were handled with multiple imputation by chained equations; results were combined using Rubin's rules. Sensitivity analyses comprised: a per-protocol analysis censoring evolocumab discontinuations; and prespecified subgroup analyses stratified by diabetes status, baseline LDL-C (≥160 vs. <160 mg/dL), and LVEF (<50% vs. ≥50%).

All statistical analyses were performed in SPSS Statistics (IBM Corp., Armonk, NY, USA). Cox proportional-hazards models used the Cox Regression procedure, logistic regression used the Binary Logistic procedure, and categorical comparisons used Fisher's exact or *χ*² tests; all tests were two-sided with *α* = 0.05.

### Ethics, registration, and role of the funder

The study received approval from the Institutional Review Board/Comitato Etico (Protocol No. 101.10.2023) and was registered with the Italian Medicines Agency (AIFA) observational registry (ID 781; code AH.011.02.023). There was no external funding; the corresponding author had full access to all the data and takes responsibility for its integrity and the accuracy of the analysis. The decision to publish was made by the investigators. The study design and outcomes were informed by contemporary guideline recommendations and evidence on early PCSK9 initiation in ACS and the long-term safety/efficacy of evolocumab (2, 5–7). With additional contextualization from real-world early-strategy implementations and CABG-specific observational data (8, 12).

## Results

We enrolled 74 ACS–CABG patients (EVOLOCUMAB *n* = 31; STANDARD *n* = 43). Of these, 71 completed 24-month follow-up and formed the analytic cohort (EVOLOCUMAB *n* = 30; STANDARD *n* = 41). Baseline characteristics were broadly balanced across groups (Table 1). Note: baseline LDL-C values reported in Table 1 refer to the enrolled cohort (*n* = 74), whereas lipid outcomes at follow-up refer to the analytic cohort (*n* = 71).

**Table 1:** Baseline characteristics.

Variable	EVOLOCUMAB (*n* = 31)	STANDARD (*n* = 43)	*p*-value
Age, years	68.5 ± 6.8	70.0 ± 7.4	NS
Male sex, *n* (%)	26 (83.9%)	34 (79.1%)	NS
Body mass index, kg/m^2^	26.9 ± 3.0	26.4 ± 2.9	NS
Diabetes mellitus, *n* (%)	12 (38.7%)	18 (41.8%)	NS
Insulin-treated diabetes, *n* (%)	7 (22.6%)	10 (23.2%)	NS
Arterial hypertension, *n* (%)	21 (67.7%)	30 (69.8%)	NS
Active smoking, *n* (%)	19 (61.3%)	25 (58.1%)	NS
Previous myocardial infarction, *n* (%)	7 (22.6%)	10 (23.2%)	0.91
Previous PCI, *n* (%)	15 (48.4%)	20 (46.5%)	NS
Previous CABG, *n* (%)	0	0	—
Peripheral arterial disease, *n* (%)	2 (6.4%)	4 (9.3%)	NS
History of stroke, *n* (%)	0	2 (4.6%)	NS
History of TIA, *n* (%)	1 (3.2%)	2 (4.6%)	NS
History of malignancy, *n* (%)	6 (19.3%)	8 (18.6%)	NS
EuroSCORE II	2.14 ± 0.75	2.05 ± 0.60	NS
Time of symptom onset, hours	98 ± 30	91 ± 27	NS
Culprit primary PCI, *n* (%)	4 (12.9%)	5 (11.6%)	NS
NSTE-ACS, *n* (%)	21 (67.7%)	30 (69.8%)	NS
STE-ACS, *n* (%)	4 (12.9%)	5 (11.6%)	NS
Unstable angina, *n* (%)	6 (19.3%)	8 (18.6%)	NS
Not on statin at baseline, *n* (%)	22 (71.0%)	32 (74.4%)	0.88
Baseline LDL-C, mg/dL	158 ± 14	154 ± 18	0.72

Of the 74 ACS patients in the study, 68 were enrolled based on LDL-C ≥ 100 mg/dL, while 6 were included at the discretion of the treating clinician due to perceived very-high-risk status, reflecting a potential selection bias and limiting generalizability to the broader ACS–CABG population.

**Figure 1:**
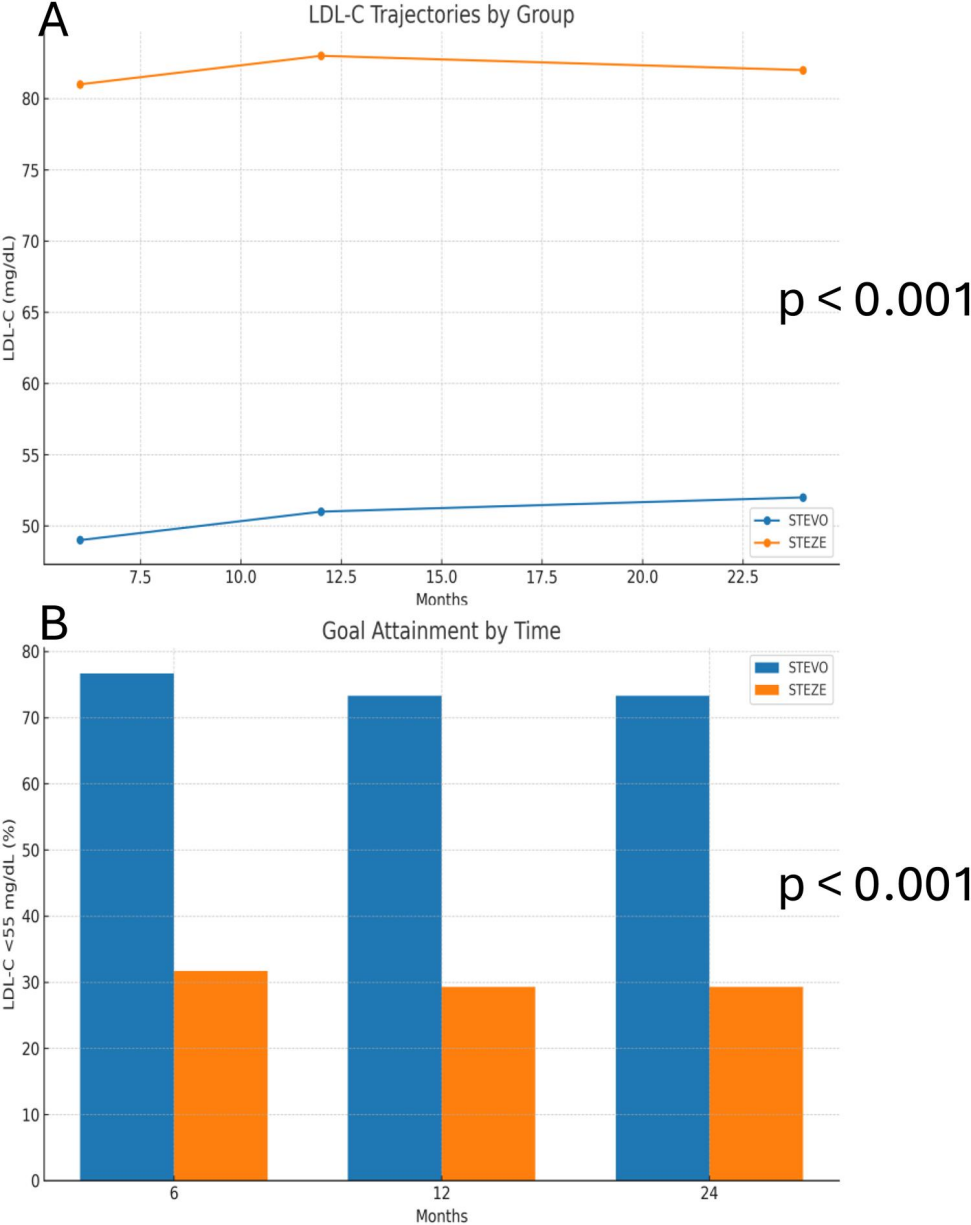
**(A)** LDL-C trajectories at 6, 12, and 24 months. All *p* < 0.001. **(B)** Proportion with LDL-C < 55 mg/dL at each time point. All *p* < 0.001.

Lipid trajectories and target attainment. LDL-C separation emerged early and persisted through 24 months. At 6 months, LDL-C was 49 ± 12 mg/dL (EVOLOCUMAB) vs. 81 ± 17 mg/dL (STANDARD); at 12 months, 51 ± 10 vs. 83 ± 19 mg/dL; and at 24 months, 52 ± 11 vs. 82 ± 18 mg/dL (all *p* < 0.001). The proportion achieving LDL-C < 55 mg/dL was 76.7% vs. 31.7% at 6 months, 73.3% vs. 29.3% at 12 months, and 73.3% vs. 29.3% at 24 months (*p* < 0.001 at each time point) (Table 2). Figure 1A shows LDL-C line plots and target attainment across time.

**Table 2:** Lipid profile at 6, 12, and 24 months.

Time	LDL-C (mg/dL) EVOLOCUMAB	LDL-C STANDARD	*p*-value	LDL-C < 55 mg/dL EVOLOCUMAB, %	LDL-C < 55 mg/dL STANDARD, %	*p*-value
6 months	49 ± 12	81 ± 17	<0.001	76.7	31.7	<0.001
12 months	51 ± 10	83 ± 19	<0.001	73.3	29.3	<0.001
24 months	52 ± 11	82 ± 18	<0.001	73.3	29.3	<0.001

**Figure 2:**
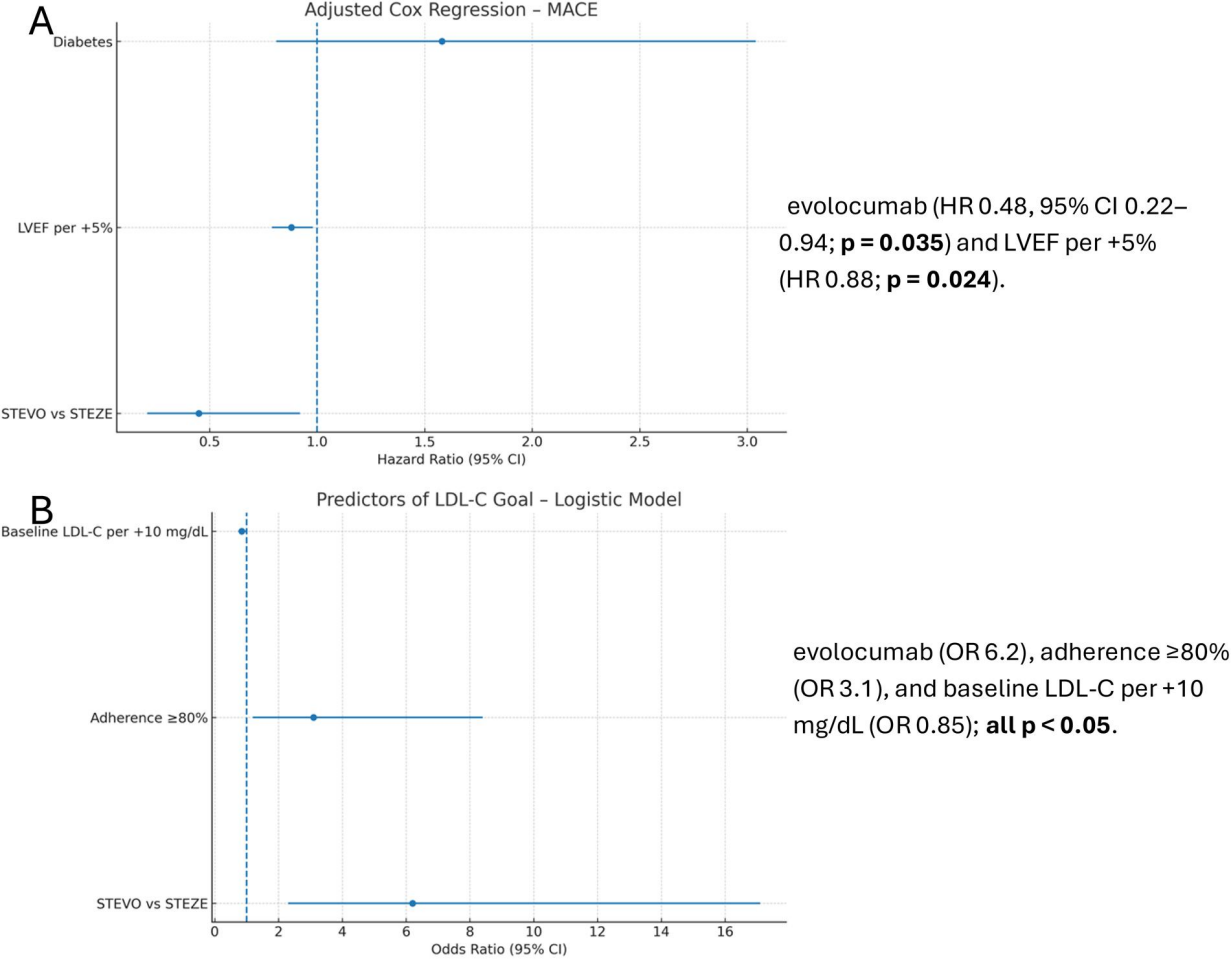
**(A)** adjusted Cox regression (MACE)—forest plot. Significant covariates: evolocumab (HR 0.48, 95% CI 0.22–0.94; *p* = 0.035) and LVEF per +5% (HR 0.88; *p* = 0.024). **(B)** Logistic regression predictors of LDL-C < 55 mg/dL—forest plot. Significant predictors: evolocumab (OR 6.2), adherence ≥80% (OR 3.1), and baseline LDL-C per +10 mg/dL (OR 0.85); all *p* < 0.05.

Primary clinical endpoint (MACE). Over 24 months, MACE occurred in 10.0% (EVOLOCUMAB) vs. 24.4% (STANDARD) (*p* = 0.035; adjusted Cox). On adjusted Cox analysis, EVOLOCUMAB was associated with a statistically significant lower risk of MACE: HR 0.48 (95% CI 0.22–0.94); *p* = 0.035 (Figure 2A; Table 3).

**Table 3:** Clinical outcomes at 24 months.

Event	EVOLOCUMAB (*n* = 30)	STANDARD (*n* = 41)	HR (95% CI)	*p*-value
MACE	3 (10.0%)	10 (24.4%)	0.48 (0.22–0.94)	0.035
Repeat revascularization	2 (6.7%)	7 (17.1%)	0.41 (0.14–0.97)	0.042
CV death	0	2 (4.9%)	–	–
Non-fatal MI	1 (3.3%)	3 (7.3%)	–	–

Secondary endpoints. Repeat revascularization occurred in 6.7% (EVOLOCUMAB) vs. 17.1% (STANDARD), with HR 0.41 (95% CI 0.14–0.97; *p* = 0.042). Rates of nonfatal MI (3.3% vs. 7.3%; *p* = 0.63) and cardiovascular death (0% vs. 4.9%; *p* = 0.51) numerically favored EVOLOCUMAB but were not individually powered (both comparisons not statistically significant). Safety outcomes were comparable between groups (the Safety subsection of Results).

Predictors of LDL-C goal attainment. In multivariable logistic regression, independent predictors of LDL-C < 55 mg/dL at 24 months were: evolocumab use (OR 6.2, 95% CI 2.3–17.1; *p* < 0.001), PCSK9inhibitor adherence ≥80% (OR 3.1, 95% CI 1.2–8.4; *p* = 0.021), and lower baseline LDL-C (OR 0.85 per +10 mg/dL, 95% CI 0.74–0.98; *p* = 0.026). Age, sex, diabetes status, and ACS type were not significant predictors (Table 4).

**Table 4:** Adjusted Cox regression for MACE and logistic regression for LDL-C < 55 mg/dL at 24 months.

Adjusted Cox regression for MACE		
Variable	HR (95% CI)	*p*-value
EVOLOCUMAB vs. STANDARD	0.48 (0.22–0.94)	0.035
LVEF per +5%	0.88 (0.79–0.98)	0.024
Diabetes	1.58 (0.81–3.04)	0.17
Logistic regression for LDL-C < 55 mg/dL at 24 months		
Variable	OR (95% CI)	*p*-value
EVOLOCUMAB vs. STANDARD	6.2 (2.3–17.1)	<0.001
PCSK9i adherence ≥80%	3.1 (1.2–8.4)	0.021
Baseline LDL-C per +10 mg/dL	0.85 (0.74–0.98)	0.026

In EVOLOCUMAB, no patient discontinued evolocumab due to adverse events. Injection-site reactions occurred in 6.7% of EVOLOCUMAB and were self-limited. No clinically significant transaminase elevations or rhabdomyolysis were observed in either group. New-onset diabetes and neurocognitive events were comparable between groups.

## Discussion

In this single-center cohort of ACS patients undergoing CABG, very-early in-hospital initiation of evolocumab on top of high-intensity statin therapy achieved rapid and durable separation of LDL-C trajectories over 24 months, with a substantially higher proportion of patients attaining guideline-recommended LDL-C targets (<55 mg/dL). This biochemical effect was accompanied by a lower incidence of major adverse cardiovascular events (MACE), primarily driven by fewer repeat revascularizations. Treatment persistence and tolerability were favorable, with no discontinuations due to adverse events and only mild injection-site reactions, supporting the feasibility of embedding PCSK9 inhibitor therapy within surgical ACS pathways.

Our findings extend the well-established benefits of PCSK9 inhibition in chronic ASCVD to the surgical ACS population. Large outcomes trials, including FOURIER, demonstrated that evolocumab added to intensive statin therapy significantly reduces MACE and drives median on-treatment LDL-C to ∼30 mg/dL, with benefits emerging early and accruing over time (5). Long-term follow-up in FOURIER-OLE showed sustained LDL-C reduction and durable safety, with evidence suggesting that earlier and longer exposure may confer incremental benefit (7). Although EVOPACS did not assess clinical outcomes, it specifically demonstrated that in-hospital initiation of evolocumab during the index ACS hospitalization is feasible, well tolerated, and achieves very high LDL-C target attainment within weeks (2). Taken together, these trials provide both mechanistic and practical justification for early PCSK9 inhibitor initiation, which is central to our strategy.

European dyslipidemia guidelines further reinforce this approach, emphasizing stringent LDL-C targets for very-high-risk patients and advocating rapid achievement of these targets following an acute event (6). In this context, early initiation may reduce the window of residual risk, a period during which patients are particularly vulnerable to recurrent ischemic events, especially in the setting of extensive multivessel or left main disease requiring surgical revascularization.

Mechanistic rationale for an “early-strike” approach is supported by multiple imaging studies. GLAGOV demonstrated that adding evolocumab to statins induces regression of coronary atheroma volume compared with statin therapy alone in patients with angiographic coronary disease (15). In the acute setting, HUYGENS, performed after non-ST-elevation myocardial infarction, showed that evolocumab plus statins increased fibrous cap thickness, reduced lipid arc, and favorably modified plaque phenotype, consistent with enhanced plaque stability (16). PACMAN-AMI tested early alirocumab after PCI for acute myocardial infarction and reported regression of nonculprit plaque burden and improvements in plaque composition at 52 weeks (17). These findings collectively support the concept that rapid and profound LDL-C lowering stabilizes vulnerable plaques across the coronary tree, a mechanism compatible with the early and persistent separation in LDL-C trajectories and the clinical event patterns observed in our cohort.

ODYSSEY OUTCOMES, although testing alirocumab in a later post-ACS window (1–12 months), demonstrated reductions in ischemic events on top of intensive statins, with signals of reduced all-cause mortality particularly among patients with higher baseline LDL-C (18). The consistency of benefit across trials and agents underscores that deep LDL-C lowering post-ACS is associated with fewer recurrent events, providing further rationale for initiating therapy even earlier, during the index hospitalization.

Operational feasibility in real-world practice is also supported by registry data. The AT-TARGET-IT registry reported that a “strike early–strike strong” PCSK9 inhibitor strategy during or immediately after ACS hospitalization is safe, practical, and achieves profound LDL-C reductions across heterogeneous high-risk populations (12). Within the surgical ACS setting, retrospective analyses indicate that early PCSK9 inhibitor use is feasible, safe, and associated with favorable lipid profiles (8). Our experience integrates these principles into a standardized CABG pathway: evolocumab was initiated before discharge, adherence was reinforced through structured follow-up, and time-to-target LDL-C was minimized compared with conventional stepwise escalation strategies.

Saphenous vein grafts (SVGs) remain particularly susceptible to early intimal hyperplasia and accelerated atherosclerosis in the first years after surgery. Although our study was not designed for systematic graft imaging, coronary CT angiography obtained in a subset of patients suggested a pattern compatible with potential SVG preservation in those receiving evolocumab. These observations, however, are exploratory and hypothesis-generating, given the non-uniform imaging protocol. Modest reductions in lipoprotein(a) achieved with PCSK9 inhibition (∼20%–25%) may also contribute to mitigating graft atherogenesis, given the atherogenic and pro-inflammatory properties of Lp(a) (19, 20). The recent NEWTON CABG trial further illustrates the complexity of SVG pathology: despite substantial LDL-C reduction, early graft disease at 24 months was not significantly altered, highlighting that factors beyond LDL-C, including local hemodynamics and biological variability, influence graft outcomes (21). Collectively, these data suggest biological plausibility but warrant careful prospective evaluation with prespecified graft patency endpoints.

Safety and tolerability in our cohort were reassuring and concordant with randomized trial data. FOURIER and FOURIER-OLE reported neutral signals regarding hepatic enzymes and muscle-related toxicity, even at very low LDL-C levels (2, 5–7). Neurocognitive safety has been rigorously assessed: the EBBINGHAUS study demonstrated no impairment in cognitive performance with evolocumab vs. placebo, and follow-up analyses reinforced absence of cognitive detriment with prolonged exposure (22, 23). Our findings—no discontinuations for adverse events and only mild injection-site reactions—mirror this evidence base, supporting the suitability of evolocumab for durable secondary prevention in surgically managed ACS.

Limitations are intrinsic to the retrospective single-center design and modest sample size, which preclude definitive causal inference and limit power for individual endpoint components such as cardiovascular death and myocardial infarction. The study is underpowered for definitive conclusions regarding hard endpoints and is primarily hypothesis-generating. Imaging ascertainment of graft patency was not performed systematically and was obtained only when clinically indicated, typically via coronary computed tomography angiography (CCTA). This non-uniform assessment limits the ability to draw definitive conclusions regarding saphenous vein graft (SVG) preservation or the impact of early LDL-C lowering on graft integrity. Consequently, any observations related to graft patency in this cohort should be considered exploratory and hypothesis-generating, and are not analyzed in detail in this report. Residual confounding cannot be excluded despite adjustment for key covariates, and unmeasured factors (e.g., socioeconomic adherence determinants) may have contributed to observed differences. Nevertheless, the internal consistency between lipid trajectories, target attainment, and downstream clinical events supports the plausibility of a treatment effect. In addition, lipoprotein(a) levels were not measured, precluding evaluation of their potential contribution to residual cardiovascular risk. Furthermore, ischemic stroke and peripheral limb events were not included among the prespecified endpoints and were not systematically collected, limiting assessment of cerebrovascular and peripheral atherosclerotic outcomes.

In summary, our results demonstrate that very-early in-hospital evolocumab initiation in ACS patients undergoing CABG is feasible, produces rapid and sustained LDL-C lowering, achieves guideline-recommended targets, and is associated with favorable clinical event patterns over two years. While graft-related observations are intriguing, they remain exploratory due to non-systematic imaging. Collectively, these findings provide a framework for integrating early PCSK9 inhibition into surgical ACS pathways, emphasizing prompt initiation, adherence support, and rapid LDL-C target attainment. Prospective multicenter studies incorporating prespecified graft patency and intravascular imaging endpoints, comprehensive Lp(a) phenotyping, and extended follow-up are warranted to determine whether early biochemical and mechanistic advantages translate into long-term reductions in hard cardiovascular endpoints in this very-high-risk surgical population.

Although our findings are encouraging, the retrospective observational design precludes definitive causal inference, residual confounding cannot be excluded, and the results remain primarily hypothesis-generating, underscoring the need for randomized trials to confirm these observations.

## Conclusions

In ACS patients undergoing CABG, early in-hospital initiation of evolocumab on top of intensive statin therapy achieved sustained LDL-C lowering and was associated with fewer adverse cardiovascular events over 24 months. As the first report in this specific population, these findings support the concept of an early, intensified lipid-lowering strategy in very high-risk surgical care. However, the study is underpowered for definitive conclusions regarding hard clinical endpoints and should be considered primarily hypothesis-generating, highlighting the need for larger, randomized trials to confirm these observations.

## Data Availability

The original contributions presented in the study are included in the article/Supplementary Material, further inquiries can be directed to the corresponding author.
